# Planned Place of Birth—Impact of Psychopathological Risk Factors on the Choice of Birthplace and Its Postpartum Effect on Psychological Adaption: An Exploratory Study

**DOI:** 10.3390/jcm11020292

**Published:** 2022-01-06

**Authors:** Clara Winter, Juliane Junge-Hoffmeister, Antje Bittner, Irene Gerstner, Kerstin Weidner

**Affiliations:** 1 Department of Psychotherapy and Psychosomatic Medicine, University Hospital Carl Gustav Carus Technische Universitaet Dresden, 01307 Dresden, Germany; Praxis@Junge-Hoffmeister.de (J.J.-H.); antje.bittner@uniklinikum-dresden.de (A.B.); Kerstin.Weidner@uniklinikum-dresden.de (K.W.); 2 Department of Gynaecology and Obstetrics, St. Elisabeth Hospital Leipzig, 04277 Leipzig, Germany; 3 Klinik Bavaria Kreischa, 01731 Kreischa, Germany; irene.gerstner@gmx.de

**Keywords:** birthplace, homebirth, birth center, mental health, pregnancy, birth anxiety, experience of violence, trauma, birth experience, postpartum psychological adaption

## Abstract

The choice of birthplace may have an important impact on a woman’s health. In this longitudinal study, we investigated the psychopathological risk factors that drive women’s choice of birthplace, since their influence is currently not well understood. The research was conducted in 2011/12 and we analyzed data of 177 women (obstetric unit, *n* = 121; free standing midwifery unit, *n* = 42; homebirth, *n* = 14). We focused antepartally (*M  =* 34.3 ± 3.3) on sociodemographic and risk factors of psychopathology, such as prenatal distress (Prenatal Distress Questionnaire), depressiveness (Edinburgh Postnatal Depression Scale), birth anxiety (Birth Anxiety Scale), childhood trauma (Childhood Trauma Questionnaire), and postpartally (*M* = 6.65 ± 2.6) on birth experience (Salmon’s Item List), as well as psychological adaption, such as postpartum depressive symptoms (Edinburgh Postnatal Depression Scale) and birth anxiety felt during birth (modified Birth Anxiety Scale). Women with fear of childbirth and the beginning of birth were likely to plan a hospital birth. In contrast, women with fear of touching and palpation by doctors and midwives, as well as women with childhood trauma, were more likely to plan an out-of-hospital birth. Furthermore, women with planned out-of-hospital births experienced a greater relief of their birth anxiety during the birth process than women with planned hospital birth. Our results especially show that women with previous mental illnesses, as well as traumatic experiences, seem to have special needs during childbirth, such as a safe environment and supportive care.

## 1. Introduction

Current international guidelines emphasize the importance for low-risk women to have a choice for their intended birthplace. In Germany, women with low-risk pregnancies may choose any birth setting, including hospital obstetric units (OU), free-standing midwifery units (FMU) or at home (homebirth; HB). Over the last ten years, the proportion of out-of-hospital births in Germany remained persistently low, at 1.5% [1]. There is evidence to suggest that hospital births are still regarded as the “standard option” that guarantees the highest possible degree of safety [2]. Furthermore, out-of-hospital birth is subject to a public controversial discourse, and women considering out-of-hospital birth are often highly criticized due to potential adverse neonatal outcome [3,4]. Studies show higher perinatal mortality and risk of neonatal morbidity in first-time mothers with planned out-of-hospital births than with planned hospital births [5]. The significantly higher technical safety in hospital obstetric units, however, contrasts higher satisfaction levels and a more positive birth experience in women with out-of-hospital births, which has important long-term implications for a mother’s health and emotional wellbeing [6,7,8,9]. It has been suggested that this positive outcome is due to the increased attention of the out-of-hospital midwives to the women’s emotional experience [7,8]. Women who experienced a planned out-of-hospital birth were more satisfied with the way their wishes and psychological demands were met, and felt significantly more involved in the decision-making processes during birth [10]. 

Women with pre-existing mental health problems and a history of traumatic experiences seem to have special needs during childbirth, such as a safe environment and supportive care [11]. However, the psychosocial factors which determine the choice of birthplace are currently not well understood. A few international studies show that pregnancy-associated anxiety, depression, or anxiety disorders are related to a preference for hospital births [12,13,14]. Except for a small German study of 74 women that could not detect a significant association between anxiety and birthplace choice (hospital vs. homebirth) [14], we are not aware of any other German study that has examined the psychopathological factors influencing choice of birthplace. To our knowledge, no study has investigated associations between childhood trauma and choice of birthplace. Epidemiological data suggest a high prevalence of childhood trauma in women, and dealing with the consequences may be a challenge in obstetric care [15]. Childhood sexual abuse is an especially traumatic event that has greater negative long-term effects on pregnant women than other physical traumas [16]. The available literature provides evidence that women with a history of childhood trauma seem to display a variety of long-term effects, such as low birthweight, re-experience of their traumatic memories by flashbacks, as well as increased anxiety and stress during labor [17,18]. Furthermore, traumatic delivery experiences are associated with an increased risk for postpartum posttraumatic stress disorder (PTSD) [19,20]. 

To ensure high quality obstetric care, the evaluation of women’s decision-making processes and women’s needs are of great importance. The knowledge of factors that influence the choice of birthplace is a prerequisite to provide specific information to pregnant women and to address individual prenatal needs. In this study, we investigated the impact of psychopathological risk factors on the planned birthplace of pregnant women. Specifically, we focused on the influence of depressive symptoms and birth anxiety, as well as childhood trauma. Postpartally, we analyzed the effects of the chosen birthplace on birth experience and psychological adaption, since this can have important implications for psychosocial and maternal postpartum adjustment [21], as well as mother–infant bonding. 

In this study, we addressed the following research questions:

1. Which psychopathological risk factors (current psychopathology, birth anxiety, childhood trauma) were associated with the choice of birthplace?
2. Does the choice of birthplace affect the birth experience of women?
3. Does the choice of birthplace influence the maternal psychopathological adjustment postpartum (i.e., postpartum depressive symptoms and birth anxiety felt during childbirth)?

## 2. Materials and Methods

### 2.1. Study Design

The current investigation was designed as a prospective longitudinal study, carried out between March 2011 and March 2012. We contacted all obstetric units, freestanding midwifery units, and freelance midwives offering antenatal classes in and around the city of Dresden, Germany. The prenatal questionnaires were distributed in antenatal classes starting from the third trimester (29th week of pregnancy). To account for the fact that less than 1.5% of all German births take place outside the hospital, we specifically contacted midwives who offered care for out-of-hospital births. Targeted oversampling allowed us to recruit a comparatively high proportion of women planning out-of-hospital births (*n* = 56 (31.6%) out of all 177). This bias by oversampling was accepted to provide special insight in this sample of women, and to make statistical comparisons feasible.

The postpartum survey was conducted by mailing a paper questionnaire with a return envelope. The data collection focused antepartally on sociodemographic factors and psychopathological risk factors, such as prenatal distress (Prenatal Distress Questionnaire; PDQ), depressiveness (Edinburgh Postnatal Depression Scale; EDPS), birth anxiety symptoms (Birth Anxiety Scale; BAS), and childhood trauma (Childhood Trauma Questionnaire; CTQ). Postpartally, the data collection (approximately 6 weeks postpartum) regarded birth experience (Salmon´s Item List; SIL) as well as psychological adaption, such as postpartal depressive symptoms (Edinburgh Postnatal Depression Scale) and birth anxiety felt during birth (modified Birth Anxiety Scale; BAS mod.). 

Study participants completed questionnaires prenatally from the 29th week of gestation (*M =* 34.3  ±  3.3, range = 29–42 weeks; hereafter defined as t_1_) and approximately 6 weeks postpartum (*M* = 6.65 ± 2.6, range = 4–20; hereafter named t_2_). The response rate at t_2_ was 88% which, in respect to the enormous changes during the postpartum adjustment process, can be considered as high. A drop-out analysis was conducted. 

All study participants gave their written consent to participate in this study. Ethical approval was granted by the Dresden University Ethics Committee (No. EK 318102010).

### 2.2. Study Participants 

Participation was voluntary and free of charge. Inclusion criteria included pregnancy in the third trimester (from 29th week) and sufficient knowledge of the German language. *n* = 177 women were recruited and participated in both assessments. The women were allocated to three major groups according to their planned birthplace with *n* = 121 in OU, *n* = 42 in FMU, and *n* = 14 in OU. At t_2_, a “congruence” variable was defined by combining the planned and actual place of birth, with three possible outcome groups, in which the planned birthplace was congruent with the actual birthplace (OU_t1=t2_, FMU_t1=t2_, HB_t1=t2_). The group of incongruent women, in which the planned birthplace (t_1_) differed from the actual birthplace (t_2_), were excluded from the postpartum analysis due to low sample size (OU_t1__≠t2_: *n* = 2; FMU _t1__≠t2_: *n* = 12, HB _t1__≠t2_: *n* = 2).

### 2.3. Instruments

The baseline questionnaire (t_1_) provided the following content: sociodemographic variables (such as age, parity, marital status, educational level, occupation, combined family income, satisfaction with financial situation), and standard questionnaires regarding potential psychopathological risk factors, in particular prenatal distress (PDQ), depressive symptoms (EPDS), birth anxiety symptoms (BAS), and childhood trauma (CTQ). The estimated processing time was 30 min. The postpartum questionnaire (t_2_) provided information on self-reported obstetric data as well as birth experience (SIL), depressive symptoms (EPDS) and birth anxiety felt during birth (BAS modified). The estimated processing time for the postpartum questionnaire was 25 min.

- Prenatal Distress

The Prenatal Distress Questionnaire (PDQ) (Yali and Lobel, 1999) measures the fears and worries of pregnant women regarding pregnancy and childbirth [22]. The twelve-item questionnaire contains self-reported fears about birth, health of the unborn child, physical changes due to pregnancy, and concerns about changes in feelings or relationships. With a possible total score of 48, a cut-off value of ≥22 is suggested. The high internal consistency of the English version is given as α = 0.81 [22].

- Depressive Symptoms

The Edinburgh Postnatal Depression Scale (EPDS) (Cox et al., 1987; German Version Bergant et al., 1998) is a widely used ten-item list for measuring depressiveness during the peripartum period with reference to the last 7 days [23,24]. Answers are rated between 0 and 3. A cut-off value of ≥10 is given, where a sum score between 10 and 12 is suggested for a moderate likelihood, and >12 for a high likelihood of a depression diagnosis. Reliability is reported to be r = 0.83 and internal consistency Cronbach’s α = 0.81 [23].

- Birth Anxiety Symptoms

The Birth Anxiety Scale (BAS) (Lukesch, 1983) is a screening instrument to assess birth-related anxiety [25]. The 77-item questionnaire contains aspects that describe situations in the birth process, self-statements about the personnel and the external environment, as well as physical and psychological stress. A cut-off value of ≥126 is suggested for an increased anxiety level. Reliability is reported to be r = 0.86 and internal consistency Cronbach’s α = 0.97 [25]. 

To uncover underlying structures of the relatively large list of birth anxiety variables, an exploratory factor analysis was computed that yielded three significant factors. Based on the factor pattern, factor 1 was labeled as “fear of birth and beginning of birth”. Here, items loaded highly included “sudden onset of labor”, fear of pain, fear of physical effort and fear of complications. Factor 2 was labeled as “fear of touching and palpation by doctors and midwives”. Items loaded highly included a general fear of doctors, nurses and midwives, as well as a fear of vaginal examinations, the gynecologist’s chair, undressing in front of the doctor, or being touched by others during labor. Factor 3 was labeled as “fear of medical procedures and clinical environment”. Here, items loaded particularly highly described fear of the clinical environment, such as noises or smell in the delivery room, and fear of medical interventions, such as the induction of labor or cesarean section (Table S1 in Supplementary Materials). Birth anxiety values related to the factor loadings of these three extracted factors were compared between the birth groups.

We modified the original BAS version for t_2_ (BAS modified) in order to retrospectively assess the actual level of birth anxiety felt during childbirth. Therefore, the individual aspects were not changed in their content.

- Childhood Trauma

The short version of the Childhood Trauma Questionnaire (CTQ) developed by Bernstein et al. (2003) (German version Wingenfeld et al., 2010) is a retrospective measure of trauma in childhood and adolescence [26,27]. It contains five subscales: emotional abuse, physical abuse, sexual abuse, emotional neglect and physical neglect. Responses are measured on a five-point Likert scale (1 = never true, to 5 = very often true). The scores are divided into four categories: none to low; low to moderate; moderate to severe; and severe to extreme trauma exposure, for each scale. Here, the categories “moderate to severe” and “severe to extreme” are considered as “presence of childhood trauma”. Reliability is reported to be r = 0.80, and internal consistency Cronbach’s α = 0.75 to 0.82 [26]. 

Additionally, we questioned whether the study participants were asked by the gynecologist or midwife about their lifetime experience of violence. If this was the case, the study participants should indicate to what extent they considered this question to be distressing.

- Birth experience

The German version of Salmon’s Item List (SIL) is a multidimensional assessment of birth experience [28]. This questionnaire is composed of 20 items, derived from terms and expressions used spontaneously by women after birth to describe their own experience. The items are rated on a numerical scale from 1 to 7, and loaded on one of the four factors: emotional adaption; physical discomfort; fulfilment; and negative emotional experience. The subscale-to-subscale correlations confirm the independence of the four dimensions ranging from 0.22 to 0.53. The Cronbach’s α-values are within an acceptable range: f1 (0.83); f2 (0.80); f3 (0.63); and f4 (0.61) [28].

### 2.4. Statistical Analysis

The statistical analyses were performed as exploratory data analyses. In addition to descriptive data analyses, a chi-square test and Fisher´s exact test were used to test the significance of differences between the birthplace groups. When the assumptions for normality of the residuals and homoscedasticity for parametric tests were met, the univariate analysis of variance (ANOVA) was used to test the association between different birthplace choices and birth anxiety symptoms (BAS), depressive symptoms (EPDS), prenatal distress (PDQ) and birth experience (SIL). The non-parametric Kruskal–Wallis H-test was used when the assumptions for parametric testing were violated. 

An exploratory factor analysis with subsequent varimax rotation was performed to identify underlying structures, or so-called factors, using the relatively large number of variables of the birth anxiety questionnaire (BAS). Using a scree-plot, the number of three latent factors to keep were selected (Figure S1 and Table S1 in Supplementary Materials). Afterwards, we applied the Kruskal–Wallis H-test to analyze the association between birthplace and latent factors. 

To identify statistically significant differences between the birthplace groups, all post-hoc analyses were α-value-corrected for multiple testing, using the Bonferroni-post hoc-method. The Bonferroni method adjusts for the Family Wise Error Rate (FWER) for multiple testing. Controlling for FWER is a very conservative approach. Thus, the Bonferroni correction enables a much stronger interpretation when results surpass this more conservative threshold [29]. 

A multinomial logistic regression model was conducted to estimate the association of prenatal distress, depressive symptoms, birth anxiety symptoms and childhood trauma (separate independent variables) with planned place of birth as the dependent variable. The associations were adjusted for potentially confounding factors. All sociodemographic factors (covariate factors) were initially included, and the model was subsequently refined by removing those factors that were not significant predictors of the outcome measure (*p* *>* 0.05). Planned hospital birth was the reference category for these comparisons.

For the postpartum comparisons of congruent groups, we compared prepartal depressiveness scores (EPDS)/birth anxiety symptoms scores during pregnancy (BAS) with postpartal depressiveness scores (EPDS)/birth anxiety felt during birth (BAS), applying a two-way ANOVA with repeated measures. The ANOVA was adjusted for the effect of time of survey on the test results, as well as interactions between time of survey and birthplace. 

All statistical analyses were carried out using SPSS Statistics Version 24.0 (IBM Corp., 2011).

## 3. Results

### 3.1. Study Population and Background Characteristics

In our study, 177 women participated in both time points (177 out of 202 women (87.6%) who were recruited at t_1_). The drop-out analysis showed statistically significant differences between women who dropped-out and those who completed the study: women who dropped out were more often primipara (X^2^ = 5.12, *p* = 0.01), less educated (X^2^ = 19.98, *p* = 0.001) and had a lower socioeconomic status (X^2^ = 11.9, *p* = 0.02), compared with the women who completed the study (Table S2 in Supplementary Materials).

At t_1_, the 177 study participants were 29 years old on average (SD 4.3 years; range 19–41) (F(2) = 0.53; *p* = 0.59) and were, on average, in their 35th week of gestation (*M  =* 34.3  ±  3.3 weeks) (Table 1). An amount of 121 women reported that they planned birth at OU (68.4%), 42 at FMU (23.7%) and 14 at home (7.9%). Of the women, 116 were primipara (65.5%) and 61 multipara (34.5%), whereby FMU and HB showed more multiparas than OU (X^2^ = 16.8, *p* < 0.001) (Table 1). Further analysis also showed that the groups differed in their occupational status (X^2^ = 31.5, *p* < 0.001). Although OU showed a relatively higher proportion of working women (86%) than the two other groups, HB showed a relatively higher proportion of housewives (31%). 

### 3.2. Psychopathological Risk Factors and Planned Place of Birth

The group comparison of the psychopathological variables resulted in nominally lower total values in HB within all tested dimensions (Table 2). Prenatal distress scores (PDQ) differed significantly between groups, which manifested in significant lower scores of HB:OU (H(2) = 14.24, *p* = 0.001) (Table 2).

**Figure 1:**
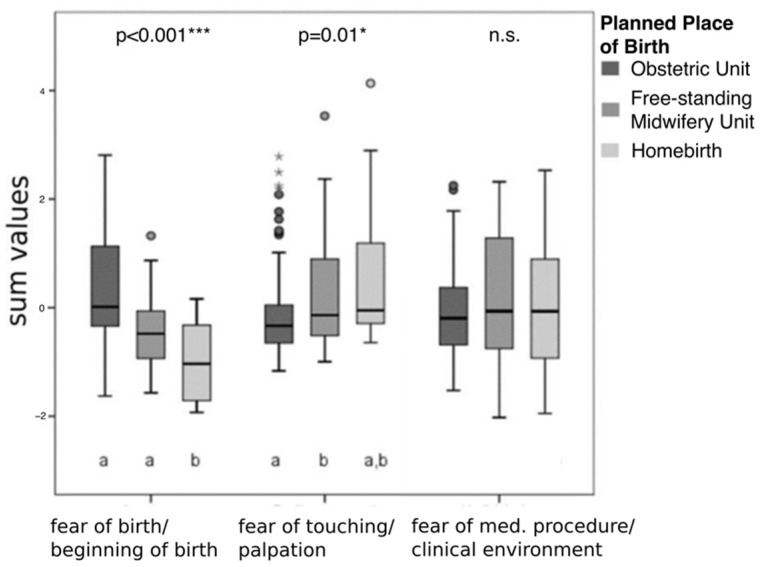
Association between planned place of birth and birth anxiety. Note: Boxplots show the median, 25%/75% quartile, whiskers with minimum/maximum as well as outliers as stars, *p* = *p*-value (* *p* < 0.05, *** *p* < 0.001, n.s. = non-significant), a/b = result of post hoc analysis to indicate statistically significant differences between the birthplace groups.

Although no group differences were found for the birth anxiety scores (BAS), the three groups differed in their patterns of anxiety (Table 2 and Figure 1). The group comparison of the three factors extracted by factor analysis showed that OU was characterized by higher relative frequencies for “fear of birth and beginning of birth” (H(2) = 29.39; *p* < 0.001), whereas FMU and HB had higher relative frequencies for “fear of touching and palpation by doctors and midwives” (H(2) = 8.53; *p* = 0.01) (Figure 1). All three birthplace groups showed similar sum values for “fear of medical procedures and clinical environment” (H(2) = 1.99; *p* = 0.37) (Figure 1). 

Childhood trauma scores (CTQ) differed between the birthplace groups (H(2) = 8.1; *p* = 0.02) with significantly higher scores in HB than OU (*p* = 0.01) (Table 2). Moreover, statistically significant differences were detected for the subscales of physical abuse and emotional neglect, that could be confirmed by post-hoc analysis for the groups HB:OU and HB:FMU (Table 2). The prevalence of childhood trauma in all women was 24.3%, and differed between the birthplace groups (H(2) = 8.7; *p* = 0.01) (Table 1). Post-hoc analysis revealed a significantly higher prevalence in HB than in OU (*p* = 0.01). Prevalence of physical abuse was statistically significantly higher in HB (38.5%) than OU (6.8%) and FMU (11.9%) (X^2^ = 9.78; *p* = 0.005) (Table 1). Although FMU women reported the highest prevalence of sexual abuse, this variable did not differ between the three birthplace groups (X^2^= 2.79; *p* = 0.19) (Table 1). Overall, a small number of three women (1.7% of all recruited women) were asked by their gynecologists (X^2^ = 3.03; *p* = 0.69) and 16 women (9.0%) by their midwives (X^2^ = 10.99; *p* = 0.003) about previous experiences of violence. In group comparison, FMU and HB were statistically significantly more frequently asked by their midwives about traumatic experiences than OU. All women reported that they found this question “not stressful” (*n* = 119; 67.2%) or “little stressful” (*n* = 58; 32.8%). 

### 3.3. Confounding Factors

A multinomial logistic regression was performed to predict the associations of psychopathological risk factors (prenatal distress, depressive symptoms, birth anxiety symptoms and childhood trauma) and choice of birthplace, after controlling for potential confounding factors. Planned hospital birth was the reference category for these comparisons. A significant regression equation was found (X^2^(10) = 58.32; *p* < 0.001), with an R^2^ of 0.36. The final model predicted 73.7% of the planned birthplaces correctly. The first set of coefficients represent the adjusted comparisons between OU and FMU. Prenatal distress (OR 0.88, 95% CI 0.81–0.95) and parity (OR 3.04, 95% CI 1.35–6.85) remained as statistically significant predictors (*p* < 0.05), indicating that women who had higher prenatal distress levels and who were primipara were more likely to choose a hospital birth (Table S3 in Supplementary Materials). The second set of coefficients represent the adjusted comparisons between OU an HB. Prenatal distress (OR 0.69, 95% CI 0.55–0.86) and childhood trauma (OR 1.15, 95% CI 1.07–1.23), as well as parity (OR 23.3, 95% CI 3.89–139.55), remained as statistically significant predictors in the model. Women who had higher prenatal distress levels, scored lower on childhood trauma, and were primipara, were more likely to choose a hospital birth than a homebirth. 

### 3.4. Birth Experience and Planned Place of Birth

The majority of women gave birth at their planned birthplace (*n* = 161), of which 119 gave birth in OU (97.5% of planned OU), 30 in FMU (73.8% of planned FMU) and 12 at home (84.6% of planned HB). Thus, for 16 women (12.5%), the actual birthplace differed from planned birthplace due to unplanned homebirth (*n* = 4), transfer to the hospital during birth (*n* = 9) and change to hospital prior to birth because of medical indications (*n* = 2).

The following analyses concerning birth experiences were conducted on the “congruent” women, for which the planned birthplace was congruent with the actual birthplace (OU_t1=t2_, FMU_t1=t2_, HB_t1=t2_). Although no group differences were found in the overall birth experience scores (SIL), the three birthplace groups differed in the dimension “good emotional adaption” (H(2) = 10.52; *p* = 0.005), which could be confirmed by a post-hoc-analysis between OU_t1=t2_:FMU_t1=t2_ (*p* = 0.04) and OU_t1=t2_:HB_t1=t2_ (*p* = 0.04) (Table 3). Thus, the congruent out-of-hospital groups showed statistically significantly better emotional adaptation during the birth process than the congruent hospital group (Table 3). Additionally, the dimension “disappointment” showed a statistically significant difference between the three groups (H(2) = 9.06; *p* = 0.01 *), which could be identified to exist between OU_t1=t2_:HB_t1=t2_ (*p* = 0.02 *) (Table 3). Thus, women of the group HB_t1=t2_ were less disappointed in their birth experience than women of the group OU_t1=t2_.

### 3.5. Postpartum Psychological Adaption and Congruent Birthplace

No differences between the congruent groups were detected for depressive symptoms (EPDS) before or after birth (t_1_: H(2) = 5.37; *p* = 0.07; t_2_: H(2) = 1.28; *p* = 0.53) (Table 4). A repeated measure two-way ANOVA, with the factors of time (2 levels) and birthplace (3 levels), showed a significant main effect of time (F(1,160) = 169.2; *p* < 0.001). The interaction of time x birthplace was non-significant (F(2,160) = 1.58; *p* = 0.21). 

When comparing prenatal birth anxiety (BAS at t_1_), the congruent groups did not show any statistically significant difference (H(2) = 0.29; *p* = 0.86) (Table 4). HB_t1=t2_ nominally reported the lowest birth anxiety during labor, whereas the sum score of OU_t1=t2_ was almost double (modified BAS at t_2_) (Table 4). The group comparison revealed a statistically significant difference between the congruent groups for birth anxiety during birth (H(2) = 19.36; *p* < 0.001), which persisted in the post-hoc analysis between OU_t1=t2_:FMU_t1=t2_ (*p* = 0.002) and OU_t1=t2_:HB_t1=t2_ (*p* = 0.014). A repeated measure two-way ANOVA with the factors of time (2 levels) and birthplace (3 levels) showed a significant main effect of time (F(1,160) = 51.34, *p* < 0.001). Accordingly, all three groups showed a statistically significant decrease in birth anxiety symptoms during the birth process. Additionally, the interaction of time x birthplace was significant (F(2;160) = 11.09; *p* < 0.001). Consequently, the out-of-hospital groups experienced a greater relief of their birth anxiety symptoms during birth process than the hospital group.

## 4. Discussion

This study presents new insights on the impact of psychopathological risk factors on the choice of birthplace. We showed that traumatic experiences as well as certain factors of birth anxiety have an impact on the choice of birthplace. Furthermore, we detected associations between the choice of birthplace and birth experience, as well as subsequent maternal psychological adaption.

### 4.1. Background Characteristics and Planned Birthplace

We identified parity as a statistically significant predictor in our regression model, indicating that women who were primiparous were more likely to choose a hospital birth. Similar results were reported in previous studies [5,10,30]. The German Society for Gynecology and Obstetrics (DGGG) clearly rejects out-of-hospital birth [31], not least due to a poorer neonatal outcome in primipara [5], which could explain the higher proportion of primipara in OU. Additionally, it can be assumed that multipara feel more self-confident, and are more likely to trust themselves with an out-of-hospital birth due to their prior birth experiences. Based on our results on the higher proportion of multiparous women in the out-of-hospital groups, we assume previous birth experiences to have a decisive influence on birthplace choice. Unfortunately, previous birth experiences were not part of the survey, and should be included in future studies. In contrast to previous studies, we could not confirm an association between a higher level of education and out-of-hospital birth [32,33,34]. Similar to the results of a Swedish case-control study with 2112 women, the out-of-hospital women were less often employed, and stayed at home more often than the hospital women in the current study [32]. It can be assumed that the out-of-hospital women represented a more family-oriented lifestyle, and that their decision to stay at home was made consciously. 

### 4.2. Psychopathological Risk Factors and Planned Place of Birth

The influence of psychopathological risk factors on the planned place of birth is currently not well understood. Witteveen et al. (2016) showed that women planning to have a hospital birth were more likely to report symptoms of depression or anxiety disorders [13]. In our study, after controlling for confounding factors, prenatal distress and childhood trauma remained statistically significant predictors in the logistic regression model. Women with higher prenatal distress level were more likely to choose a hospital birth. In all measured psychopathological risk factors, HB showed the lowest nominal scores, which indicated a lower psychopathological burden, overall. Looking at the total score of birth anxiety solely, there was no statistically significant difference between our three groups. This contrasts with existing studies, that reported higher levels of birth anxiety for women that chose a hospital birth [12,13]. Witteveen et al. (2016) observed significantly higher levels of birth anxiety among clinical women with a comparatively higher risk awareness, and a stronger need for obstetric safety [13]. In contrast to our detected total scores, we could reveal different birth anxiety contents between the groups on individual categories. OU´s birth anxiety contents centered mainly on “fear of childbirth and beginning of birth” whereas, in contrast, the fear of our two out-of-hospital-groups referred to “fear of touching and palpation by doctors and midwives”. 

Overall, about one quarter of all study participants reported childhood trauma. HB was especially associated with higher prevalence of early traumatic experiences. They reported more statistically significant occurrences of physical abuse and emotional neglect than the other two groups. A history of childhood trauma can have a variety of long-term effects; it increases vulnerability for low birthweight, as well as flashbacks due to the re-experience of traumatic memories, and increased anxiety and stress during labor [17,18]. Pregnant women with previous experiences of traumatic violence have a need for familiar surroundings, trusted personnel, and active and self-determined participation in the birth process in order to feel in control during childbirth [11]. These aspects are more likely to be attributed to out-of-hospital births [7,8]. It is essential to identify women with previous experiences of violence at an early stage in pregnancy, in order to take the special needs during childbirth into account. However, due to shame and insecurity, affected women usually do not talk about their experiences of traumatic violence of their own accord [35,36]. Only 1.7% of all study participants were asked by their gynecologists, and 9.0% by midwives, about their traumatic experiences, although this question was not perceived as stressful by the women surveyed. The out-of-hospital groups were asked significantly more often by their midwives about previous trauma than the hospital group. The holistic care approach of the out-of-hospital midwives, that allows them to know the women at an early stage and accompany them through the entire pregnancy, could explain such thoughtful and careful care. Healthcare professionals may feel insufficiently prepared for dealing with traumatized women [35]. Therefore, medical personnel should be made more aware of this issue and its potential effects on pregnancy and childbirth, as well as the postpartum adaption. Many authors called for a screening of all women for the experience of traumatic violence [35,37]. Applying such suggested screening could facilitate the identification of affected pregnant women, and could help to provide them with optimal obstetric care. The available resources for optimal care of traumatized women could comprise, e.g., sensitive, individual birth preparation, postpartum monitoring, (trauma) psychotherapy if necessary, information about self-determination in clinical settings, building a relationship with the child, and learning stabilization techniques and trigger identification [35]. Continuous care during childbirth can help to avoid traumatizing birth processes [38]. However, since not all affected pregnant women can be identified with certainty, all women should receive trauma-sensitive care during childbirth, in order to prevent trauma and re-traumatization [39]. A study by Bohren et al. (2017) revealed that continuous care of pregnant women during childbirth could improve maternal and neonatal outcome, due to a higher spontaneous birth rate and a shorter duration of labor [40].

### 4.3. Birth Experience, Postpartal Psychological Adaption and Congruent Birthplace

As mentioned above, care concepts of out-of-hospital births provide continuous care by the same person during pregnancy and the postpartum period, as well as one-to-one care during childbirth. This intensive care concept was identified as positive influencing factor on birth experience [6,7,8]. Contrasting these study results, we could not identify a significant difference in the overall level of birth experience between our congruent groups. Still, the two congruent out-of-hospital groups showed statistically significant better emotional adaptation during the birth process than the congruent OU. Additionally, the congruent HB was statistically significantly less disappointed in their birth experience than the congruent OU. It can be assumed that high-risk pregnancies are more frequent in OU (data concerning pregnancy risks was not available) and affected women need to be monitored more closely during childbirth, which may hamper an undisturbed birth experience and promote a higher rate of operative termination of labor. Furthermore, the congruent out-of-hospital groups experienced greater relief of their birth anxiety during the birth process than OU. An increased focus of out-of-hospital midwives on the psychological dimension during childbirth was also reported as a reason for the higher satisfaction in out-of-hospital births [7,8]. A pronounced feeling of control, emotional support, and a sense of empowerment by the midwife were also mentioned to have a positive effect on childbirth [41]. Conclusively, we identified an association between choice of birthplace and birth experience, most probably due to the different care concepts. To prevent traumatic birth outcomes, continuous care during labor can improve the emotional security of childbearing women, regardless of the birthplace. 

### 4.4. Limitations of the Research Design

A possible systematic bias due to the recruitment of pregnant women via birth preparation courses can be assumed. It is possible that women with mental illnesses (such as social anxiety) tend to avoid participation in prenatal classes and may be underrepresented in this study. Furthermore, women who were less educated and had lower regular income were less likely to complete the study. In future studies, it would be desirable to encourage more of these women to participate. A selection bias was accepted by targeted oversampling of women with planned out-of-hospital birth, which ultimately resulted in neither an equal distribution of the groups nor a representative sample of the true population. Thus, a comparatively high proportion of women with planned out-of-hospital birth (31%) could be recruited, which enabled a special view on this group of women. The most important limitation of our research design is, however, the small subsample of 14 women in HB, since this hampers the extrapolation on a larger scale. The fact that the proportion of out-of-hospital births in Germany has remained persistently low at 1.5% demonstrates the difficulty to recruit women with planned homebirth. It should be noted that the data were collected in 2011/2012. However, the psychosomatic content of the German maternity guidelines (so-called “Mutterschaftsrichtlinien”) has not changed substantially since then, so it is reasonable to assume that a similar study would reach the same results today. 

Although the associations between the tested risk factors of psychopathology and planned place of birth were adjusted for potential confounding factors, it remains possible that there are additional confounding variables that were not included in the models. It can be assumed that previous birth experiences may have a decisive influence on the choice of birthplace, but this was not part of the survey. We suggest that future birthplace studies include previous birth experiences in multiparous women to be able to control for such potential confounding factors. 

To minimize the time effort for the women, we kept the questionnaire as short as possible. We had to accept a further possible bias in using self-reporting questionnaires to record women´s sociodemographic data. Additionally, it must be critically discussed that all evaluated data were subject to women´s self-assessment and thus could be influenced by subjective views. However, numerous validated psychometric questionnaires were used that showed good psychometric properties. Whereas the German version of the EPDS has been shown to have a good reliability [24], we still lack a German validation of EPDS in pregnancy. Still, the applicability of the original EPDS for use during pregnancy has been well established [42].

Furthermore, we had no access to the participants’ medical records which could facilitate the identification of specific medical implications for a certain birthplace, especially for the OU group. Future research should address other mental health conditions such as anxiety disorders or PTSD, as well as other traumatic events such as intimate partner violence or traumatic birth experience, since these have not been under investigation so far. We will only be able to provide optimal care if we are aware about the special requirements of affected women for their planned place of birth. 

## 5. Conclusions

Our study demonstrated that psychopathological risk factors and traumatic experiences had an impact on the decision-making process of pregnant women for their choice of birthplace. We showed that factors such as birth anxiety and prenatal distress influenced the choice of birthplace, and that women with early traumatic experiences were significantly more likely to choose an out-of-hospital birth. 

Additionally, our study demonstrated that the choice of birthplace influenced the birth experience and maternal psychological adjustment. A deeper understanding of pregnant women’s needs will help us to individually adapt birth planning, birth talks, and care during childbirth. A routine screening on traumatic experiences should be included in prenatal care to provide affected women with optimal obstetric care, to prevent re-traumatization and to improve their childbirth experience. Regardless of the choice for a planned hospital or out-of-hospital birth, continuous care can help to improve for the emotional security of pregnant women in order to create a trusting, secure environment, which is an important prerequisite to prevent traumatic birth outcomes. Psychopathological risk factors and traumatic experiences should be adequately addressed in midwifery and gynecological routine examination for an informed decision-making process regarding the planned place of birth.

## Figures and Tables

**Table 1:** Background characteristics of *n* (%) in the total sample, according to planned birthplace.

Variables	Total	OU	FMU	HB	Test Stat.	*p*
Age (years) ^1^	29.1 ± 4.3	29.1 ± 4.0	28.9 ± 4.9	30.3 ± 4.8	F = 0.53	0.59
Parity					X^2^ = 16.8	<0.001 **
Primipara	116 (65.5%)	91 (74.4%)	22(52.4%)	3 (23.1%)		
Multipara	61 (34.5%)	30 (25.6%)	20 (47.6%)	11 (76.9%)		
Marital Status					X^2^ = 11.7	0.34
Married	72 (41.6%)	42 (35.6%)	21 (50%)	9 (69.2%)		
Permanent relationship	93 (53.8%)	70 (59.3%)	19 (45.2%)	4 (30.8%)		
Single	5 (2.9%)	3 (2.5%)	2 (4.8%)	-		
Living separately	1 (0.6%)	1 (0.8%)	-	-		
Other	1 (0.6%)	1 (0.8%)	-	-		
Education Level					X^2^ = 10.7	0.33
Middle-school till 9th grade	2 (1.2%)	-	1 (2.4%)	1 (7.7%)		
Middle school till 10th grade	36 (20.8%)	27 (22.9%)	8 (19.0%)	1 (7.7%)		
High-school diploma	75 (44.5%)	50 (42.4%)	17 (40.5%)	8 (61.5%)		
University and masters	60 (33.5%)	41 (34.7%)	16 (38.1%)	3 (23.1%)		
Occupation					X^2^ = 31.5	<0.001 ***
Employed	136 (76.8%)	101 (85.6%)	26 (61.9%)	6 (46.2%)		
Housewife	6 (3.5%)	1 (.8%)	1 (2.4%)	4 (30.8%)		
In education	17 (9.8%)	7 (5.9%)	9 (21.4%)	1 (7.7%)		
Unemployed	10 (5.8%)	5 (4.2%)	3 (7.1%)	2 (15.4%)		
Other	7 (4.0%)	4 (3.4%)	3 (7.1%)	-		
Combined Family Income (per annum)					H = 3.0	0.22
<6000€	5 (2.9%)	1 (0.9%)	3 (7.3%)	1 (7.7%)		
6000–12,000€	19 (11.0%)	9 (7.7%)	7 (17.1%)	3 (23.1%)		
12,000–18,000€	25 (14.5%)	16 (13.7%)	8 (19.5%)	1 (7.7%)		
18,000–24,000€	31 (17.9%)	26 (22.2%)	4 (9.8%)	1 (7.7%)		
24,000–36,000€	54 (31.2%)	40 (34.2%)	11 (26.8%)	3 (23.1%)		
>36,000€	37 (21.4%)	25 (21.4%)	8 (19.5%)	4 (30.8%)		
Satisfaction with Financial Situation					X^2^ = 8.2	0.19
Satisfied	59 (34.1%)	43 (36.8%)	9 (21.4%)	7 (53.8%)		
Rather satisfied	33 (19.1%)	21 (17.9%)	9 (21.4%)	3 (23.1%)		
Rather unsatisfied	71 (41.0%)	45 (38.5%)	23 (54.8%)	3 (23.1%)		
Unsatisfied	9 (5.2%)	8 (6.8%)	1 (2.4%)	-		
Childhood Trauma (CTQ)	42 (24.3%)	22 (18.6%)	13 (31.0%)	7 (53.8%)	X^2^ = 8.7	0.01 *
Physical abuse	18 (10.4%)	8 (6.8%)	5 (11.9%)	5 (38.5%)	X^2^ = 9.8	0.005 **
Sexual abuse	19 (11.0%)	10 (8.5%)	7 (16.7%)	2 (15.4%)	X^2^ = 2.8	0.19
Emotional neglect	9 (5.2%)	6 (5.1%)	1 (2.4%)	2 (15.4%)	X^2^ = 3.1	0.19
Physical neglect	6 (3.5%)	5 (4.2%)	1 (2.4%)	-	X^2^ = 0.27	>0.99
Emotional abuse	27 (15.6%)	20 (16.9%)	4 (9.5%)	3 (23.1%)	X^2^ = 2.0	0.39

Note: data in *n* (%), ^1^ in mean (SD), OU = obstetric unit, FMU = free-standing midwifery unit, HB = homebirth, CTQ = Childhood Trauma Questionnaire (categories “moderate to severe” and “severe to extreme” are considered to be “presence of childhood trauma”), X^2^ = Fisher’s exact test, H = Kruskal–Wallis H-test, *p* = *p*-value (* *p* < 0.05; ** *p* < 0.01; *** *p* < 0.001).

**Table 2:** Association between planned place of birth and prepartal scores of prenatal distress, depressive symptoms, birth anxiety symptoms and childhood trauma (t_1_).

		*n*	Mean	SD	Min	Max	H	*p*
(t_1_) PDQ	OU	121	14.5	6.9	3	31	14.24	0.001 **
	FMU	42	12.1	7.9	2	27		
	HB	14	7.9	5.3	3	20		
(t_1_) EPDS	OU	121	5.8	3.8	0	18	3.7	0.16
	FMU	42	6.7	4.5	0	17		
	HB	14	4.4	4.7	0	17		
(t_1_) BAS	OU	121	75.3	34.7	9	178	1.35	0.51
	FMU	42	77.9	38.8	15	170		
	HB	14	61.7	36.5	0	139		
(t_1_) CTQ	OU	121	11.43	2.99	10	25	8.09	0.02 *
	FMU	42	12.37	4.80	10	32		
	HB	14	15.15	5.58	10	27		
Physical abuse	OU	121	5.97	2.42	5	20	13.17	0.001 **
	FMU	42	5.74	1.85	5	13		
	HB	14	9.62	5.32	5	22		
Sexual abuse	OU	121	5.47	1.39	5	11	3.39	0.18
	FMU	42	6.22	3.12	5	20		
	HB	14	6.54	3.46	5	16		
Emotional abuse	OU	121	8.49	4.19	5	21	8.01	0.02 *
	FMU	42	8.12	3.05	5	19		
	HB	14	11.46	4.27	5	19		
Physical neglect	OU	121	5.83	1.48	4	10	1.14	0.57
	FMU	42	5.89	1.61	4	11		
	HB	14	6.23	1.59	5	9		
Emotional neglect	OU	121	6.92	3.14	5	20	2.77	0.25
	FMU	42	6.76	2.66	4	16		
	HB	14	8.23	4.02	5	18		

Note: OU = obstetric unit, FMU = free-standing midwifery unit, HB = homebirth, PDQ = Prenatal Distress Questionnaire (Cut-off ≥ 22), EPDS = Edinburgh Postnatal Depression Scale (Cut-off ≥ 10; 10–12 = moderate; >12 = high likelihood of a depression), BAS = Birth Anxiety Scale (Cut-off ≥ 126); CTQ = Childhood Trauma Questionnaire, H = Kruskal–Wallis H-test, *p* = *p*-value (* *p* < 0.05; ** *p* < 0.01).

**Table 3:** Association between birth experience and actual birthplace, for women who gave birth at planned birthplace.

SIL	Group	*n*	Mean	SD	Min	Max	H	*p*
Total score	OU_t1=t2_	119	82.8	20.5	23	114	3.47	0.18
	FM_t1=t2_	30	87.7	20.5	34	112		
	HB_t1=t2_	12	93.3	8.4	78	105		
Fulfilment	OU_t1=t2_	119	33.4	8.8	6	42	0.68	0.71
	FM_t1=t2_	30	34.4	8.6	7	42		
	HB_t1=t2_	12	35.0	3.9	27	40		
Good emotional adaption	OU_t1=t2_	119	26.0	7.0	6	35	10.52	0.005 **
	FM_t1=t2_	30	28.2	7.7	8	35		
	HB_t1=t2_	12	30.7	3.4	24	35		
Negative emotional experience ^1^	OU_t1=t2_	119	18.8	3.6	6	21	9.06	0.01 *
	FM_t1=t2_	30	19.3	3.4	10	21		
	HB_t1=t2_	12	20.9	0.3	20	21		
Physical discomfort ^1^	OU_t1=t2_	119	11.3	3.8	3	21	0.75	0.69
	FM_t1=t2_	30	11.7	4.1	4	20		
	HB_t1=t2_	12	12.2	2.9	8	18		

Note: ^1^ Note the converted polarity of the subscales “negative emotional experience” and “physical discomfort”, SIL = German version of Salmon’s Item List (Cut-off ≥ 70), t1 = t2: planned birthplace congruent with actual birthplace: OU = obstetric unit, FMU = free-standing midwifery unit, HB = homebirth, SD = standard deviation, Min = minimum, Max = maximum, H = Kruskal–Wallis H-test, *p* = *p*-value (* *p* < 0.05; ** *p* < 0.01).

**Table 4:** Association between depressive symptoms, birth anxiety scores and actual birthplace of women who gave birth at planned birthplace (antepartal (t_1_) and postpartal (t_2_)).

		*n*	Mean	SD	Min	Max	H	*p*
(t_1_) EPDS	OU_t1=t2_	119	5.85	3.92	0	18	5.37	0.07
	FM_t1=t2_	30	6.57	4.69	0	17		
	HB_t1=t2_	12	4.50	4.90	0	17		
(t_2_) EPDS	OU_t1=t2_	119	11.01	3.26	7	23	1.28	0.53
	FM_t1=t2_	30	11.53	3.34	7	19		
	HB_t1=t2_	12	11.70	4.52	7	19		
(t_1_) BAS	OU_t1=t2_	119	75.50	34.34	9	178	0.29	0.86
	FM_t1=t2_	30	77.67	39.83	15	170		
	HB_t1=t2_	12	78.50	32.59	26	139		
(t_2_) BAS ^1^	OU_t1=t2_	119	66.40	32.49	4	176	19.36	<0.001 ***
	FM_t1=t2_	30	44,43	20.47	12	99		
	HB_t1=t2_	12	36.90	23.12	6	66		

Note: ^1^ Note the modified version of BAS for the postpartum period to record retrospectively the actual level of birth anxiety symptoms during birth; t1 = t2: planned birthplace congruent with actual birthplace: OU = obstetric unit, FMU = free-standing midwifery unit, HB = homebirth, EPDS = Edinburgh Postnatal Depression Scale (Cut-off ≥ 10–12 = moderate; >12= high likelihood of a depression), BAS = Birth Anxiety Scale (Cut-off ≥ 126); SD = standard deviation, min = minimum, max = maximum, H = Kruskal–Wallis H-test, *p* = *p*-value (*** *p* < 0.001).

## Data Availability

The data presented in this study are available on request from the corresponding author.

## Supplementary Materials

The following supporting information can be downloaded at: https://www.mdpi.com/article/10.3390/jcm11020292/s1, Figure S1: Screeplot for factor analysis: 3 latent factors were selected to keep; Table S1: Background characteristics in *n* (%) and drop-out-analysis; Factor loadings after exploratory factor analysis for Birth Anxiety Scale (BAS); Table S2: Background characteristics in *n* (%) and drop-out-analysis; Table S3: Logistic Regression Model of Predictors of Planned Birthplace.
