# Supplementary material for: Planned Place of Birth—Impact of Psychopathological Risk Factors on the Choice of Birthplace and Its Postpartum Effect on Psychological Adaption: An Exploratory Study
Source: J Clin Med. 2022 Jan 6;11(2):292. doi: 10.3390/jcm11020292 (PMC8779342; doi:10.3390/jcm11020292)
Supplement: Supplementary file 1 [file jcm-11-00292-s001.zip › jcm-1520402-supplementary.pdf]

Journal of
Clinical Medicine

MDPI

Supplementary Materials

Exploratory Factor Analysis of Birth Anxiety Scale (BAS)

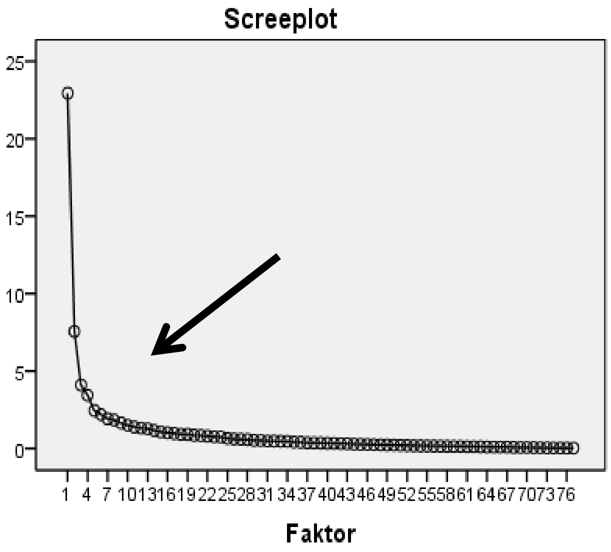

Figure S1. Screeplot for factor analysis: 3 latent factors were selected to keep.

Table S1. Factor loadings after exploratory factor analysis for Birth Anxiety Scale (BAS).

Rotated component matrix			
	Factors		
**Items**	**I**	**II**	**III**
General Anxiety that birth is imminent		0.734	
Fear of gynaecological examinations (birth context)			0.695
Fear of gynaecological examinations in general (independent of pregnancy)			0.785
Fear of gynaecologist			0.716
Fear of gyn chair			0.774
Fear of going to and from the gyn chair			0.770
Fear of undressing at the gynaecologist			0.736
Fear of being looked at by the gynaecologist			0.769
Pain during examination			0.580
Injections	0.563		
Surgical instruments	0.622		
Open wounds on myself	0.517		
Open wounds on others	0.569		
Vomiting			
Nausea		0.403	
Fear of losing the child, i.e. having a miscarriage			
Fear of unexpected or sudden onset of childbirth		0.674	
Fear of the beginning of labor		0.820	

Rotated component matrix			
		Factors	
Items	I	II	III
Departure from home to birth place		0.666	
Entering the birth place		0.456	
Entering the admission room	0.422	0.414	
Noises in the hospital	0.608		
Sick people	0.624		
Watching others getting an injection	0.497		
Watching others getting prepared for birth process	0.510		
Vaginal examinations during birth process			0.436
Fear of taking medications yourself			
Syringes and sharp objects such as knives, scissors and scalpels	0.656		
Fear of contractions		0.756	
Fear of being touched			0.564
Injections for birth induction	0.648		
Iv drip for birth induction	0.616		
Waiting in admission room for birth	0.530		
Getting an enema before birth	0.480		
Shaving and bathing	0.443		0.471
Being brought to delivery room	0.554		
Fear of entering delivery room	0.567	0.407	
Fear of the bright lamps above the gynaecologist chair	0.595		0.411
Fear of odors in delivery room	0.716		
Fear of noises in delivery room	0.768		
Instruments and equipment in delivery room	0.797		
Fear of the medical doctors in delivery room	0.649		0.525
Fear of young medical doctors	0.540		0.485
Fear of nurses	0.487		0.582
Fear of midwives			0.505
fear of unfriendly and impatient people in the delivery room	0.437		
Mass processing and routine treatment	0.523		
Fear of fear of rupture of membranes		0.628	
Of long duration of birth		0.727	
Of complications		0.675	
Fear of not being able to endure the effort		0.764	
Fear of the expulsion period		0.771	
Fear of losing urine and flatulences when pressing		0.675	
Fear of pain		0.796	
Fear of blood of yourself	0.422	0.434	
Fear of blood of others	0.559		
Fear of being left alone			
Fear of losing self-control		0.528	
Fear of losing nerves		0.644	
Fear of hearing yourself screaming		0.551	
Fear of hearing others screaming	0.437		
Fear of being poorly prepared for birth		0.585	
Fear of not being educated enough about the birth process		0.571	
Fear of being looked at by people standing around	0.417		0.422
Fear of Anesthesia	0.758		
Fear of cesarean section	0.634		
Fear of being seen half naked by others during birth			0.484

Rotated component matrix			
		Factors	
Items	I	II	III
Fear of not knowing what is happening to you and why	0.426		
Fear of feeling oppressed	0.401	0.482	0.403
Fear of episiotomy		0.466	
Fear of the suture of an episiotomy	0.423	0.440	
Feeling of hopelessness	0.463	0.430	
Fear of your own death			
Fear of being exposed			
Fear of afterbirth		0.518	
Fear of the first sight of the child			
Fear of congenital anomalies		0.553	

Note: Exploratory factor analysis was performed by administering 77-items questionnaire to 177 study participants. The Kaiser–Meyer–Olkin measure of sampling adequacy was .85, representing a relatively good factor analysis, and Bartlett's test of Sphericity was significant (p<0.001), indicating that correlations between items were sufficiently large for performing an EFA. Using scree-plot 3 latent factors were selected to keep, which accounted for 44.95% of the total variance. Factor I was interpreted as „ fear of medical procedures and clinical environment", Factor II as „fear of birth and beginning of birth" and Factor III as „fear of physical contact and palpations".

Table S2: Background characteristics in *n* (%) and drop-out-analysis

Variables	Total	Drop-outs	Completer	Test stat.	*p*
parity				X^2=5.12	0.009**
primipara	71 (35.3%)	11 (44.0%)	60 (34.1%)		
multipara	130 (64.7%)	14 (56.0%)	116 (65.9%)		
marital status				X^2=3.39	0.51
married	80 (39.6%)	7 (28.0%)	73 (41.2%)		
permanent relationship	114 (56.4%)	17 (68.0%)	97 (54.8%)		
single	6 (3.0%)	1 (4.0%)	5 (2.8%)		
living separately	1 (0.5%)	-	1 (0.6%)		
other	1 (0.5%)	-	1 (0.6%)		
education level				X^2=19.98	0.001**
middle-school till 9th grade	6 (3.0%)	4 (16.0%)	2 (1.1%)		
middle-school till 10th grade	48 (23.8%)	10 (40.0%)	38 (21.5%)		
high-school diploma	83 (41.0%)	7 (28.0%)	76 (43.0%)		
university and master	62 (30.7%)	3 (12.0%)	59 (33.3%)		
other	3 (1.5%)	1 (4.0%)	2 (1.1%)		
occupation				X^2=1.88	0.94
employed	156 (77.2%)	20 (80.0%)	136 (76.8%)		
unemployed	13 (6.4%)	2 (8.0%)	11 (6.2%)		
housewife	7 (3.5%)	1 (4.0%)	6 (3.4%)		
in education	18 (8.9%)	1 (4.0%)	17 (9.6%)		
other	8 (4.0%)	1 (4.0%)	7 (4.0%)		
combined family income (per annum)				X^2=11.9	0.02
<6000€	7 (3.5%)	2 (8.0%)	5 (2.9%)		
6000-12000€	25 (12.5%)	5 (20.0%)	20 (11.4%)		
12000-18000€	31 (15.5%)	5 (20.0%)	26 (14.9%)		

Variables	Total	Drop-outs	Completer	Test stat.	*p*
18000-24000€	39 (19.5%)	8 (32.0%)	31 (17.7%)		
24000-36000€	58 (29.0%)	2 (8.0%)	56 (32.0%)		
>36000€	40 (20.0%)	3 (17.0%)	37 (21.1%)		
satisfaction with financial situation				X^2=6.21	0.09
satisfied	65 (32.3%)	5 (20.0%)	60 (34.1%)		
rather satisfied	44 (21.9%)	10 (40.0%)	34 (19.3%)		
rather unsatisfied unzufrieden	81 (40.3%)	8 (32.0%)	73 (41.5%)		
unsatisfied	11 (5.5%)	2 (8.0%)	9 (5.1%)		

Note: X^2= Fisher´s exact test, *p*=*p*-value (** $p<0.01$)

Table S3. Logistic Regression Model of Predictors of Planned Birthplace.

Birthplace	Predictor	B	*p*-Value	Odds Ratio	95% Confidence Interval	
					Lower Bound	Upper Bound
FMU	PDQ	−0.13	0.001**	0.88	0.81	0.95
	EPDS	0.07	0.19	1.08	0.97	1.19
	BAS	0.01	0.08	1.01	0.99	1.03
	CTQ	0.03	0.19	1.03	0.99	1.07
	Parity	1.11	0.007**	3.04	1.35	6.85
HB	PDQ	−0.38	0.001**	0.69	0.55	0.86
	EPDS	−0.06	0.58	0.94	0.76	1.16
	BAS	0.02	0.21	1.02	0.99	1.04
	CTQ	0.14	<0.001***	1.15	1.07	1.23
	Parity	3.15	0.001**	23.29	3.89	139.55

Note: A multinomial logistic regression model was conducted to estimate the association of prenatal distress, depressive symptoms, birth anxiety and childhood trauma (separate independent variables) with planned place of birth as dependent variable. The associations were adjusted for potentially confounding factors. All sociodemographic factors (covariate factors) were initially included, and the model was subsequently refined by removing those factors that were not significant ($p>0.05$) predictors of the outcome measure. Planned hospital birth was the reference category for these comparisons.

(OU=Obstetric Unit, FMU=Free-standing Midwifery Unit, HB=Homebirth, PDQ= Prenatal Distress Questionnaire (Cut-off≥22), EPDS=Edinburgh Postnatal Depression Scale (Cut-off ≥10–12=moderate; >12= high likelihood of a depression), BAS=Birth Anxiety Scale (Cut-off ≥126); CTQ=Childhood Trauma Questionnaire, B= coefficient, *p*-value (** $p<0.01$; ***$p<0.001$)).
